# Inhibition of JK184-Induced Cytoprotective Autophagy Potentiates JK184 Antitumor Effects in Breast Cancer

**DOI:** 10.1155/2020/1657896

**Published:** 2020-06-02

**Authors:** Xiaoya Xi, Tao He

**Affiliations:** Institute for Cancer Medicine and School of Basic Medical Sciences, Southwest Medical University, Luzhou, Sichuan 646000, China

## Abstract

Breast cancer (BCa) is the most common aggressive tumor with limited curative therapeutic options available among women worldwide. JK184 is a potent Hedgehog inhibitor that regulates the glioma-dependent transcriptional activity. Although some studies have indicated that JK184 can kill BCa cells, it remains unclear whether there are any events that limit the use of JK184 in BCa therapy. Here, we report that JK184 intervention induces BCa cell death involving the dysregulation of autophagy in a dose- and time-dependent manner. The induction of autophagy compromises the antiproliferative effect of JK184. Mechanistically, JK184 induces autophagy via inhibiting the Akt/mTOR pathway in BCa cells. Taken together, our findings unravel a novel mechanism for JK184 treatment in BCa, suggesting that JK184 in combination with autophagy inhibitor may be a potential therapeutic strategy for the clinical treatment of BCa.

## 1. Introduction

Breast cancer is the most diagnosed cancer in women, accounting for about 15% of all new cases [1]. The morbidity and mortality of BCa are expected to rise significantly in the next decades. In recent decades, the strategy of surgical resection combined with radiotherapy has afforded effective treatment outcomes for early or local disease [2]. However, most patients are diagnosed in advanced stages and require cytotoxic chemotherapy, endocrine therapy or combinational therapy. Due to drug resistance or cancer recurrence, the current therapeutic strategies for BCa display compromised treatment outcome, and the overall prognosis for BCa patients remains poor. Therefore, there is an urgent need to develop novel chemotherapeutic drugs for the treatment of BCa patients [3].

Autophagy is a deeply conserved homeostatic pathway and plays pivotal roles in the inhibition of tumorigenesis [4]. However, once the tumor is formed, autophagy enables tumor cells to survive stresses in the microenvironment, contributing to cancer progression [5]. Recently, the Nobel Prize in Physiology or Medicine has been awarded to Yoshinori Ohsumi for his work regarding the autophagy process [6]. Notably, the dysregulation of autophagy has been indicated as a cause of resistance to therapies [7, 8]. For example, treatment of BCa cells with doxorubicin could trigger autophagy to inhibit doxorubicin-mediated apoptosis and restore growth inhibition [9]. Autophagy may play a role in mediating programmed cell death. A recent study has demonstrated that itraconazole, a common antifungal agent, exhibits antiproliferation activity against BCa cells through inducing apoptosis and autophagic cell death [10]. As such, the underlying mechanisms of autophagy in BCa cells remain poorly characterized. Accordingly, autophagy is an actionable target for improving therapy efficacy in BCa following identification of the biological roles of autophagy in treatment.

JK184 is a selective Gli inhibitor that antagonizes the Hedgehog pathway via inhibition of glioma-dependent transcriptional activation of target genes. Previous studies have reported that JK184 induces apoptotic cell death and inhibits the growth of Panc-1 and BxPC-3 cells both *in vitro* and *in vivo* [11]. However, whether any additional pathways are involved in the antitumor activity of JK184 remains to be further defined. Furthermore, there are rarely reports for JK184 in growth suppression of BCa cell and the limitation of JK184 in BCa therapy, such as the off-target effect.

In this study, we demonstrate that JK184 significantly inhibits the growth of BCa cells. The induction of complete autophagic flux is characterized as the key event in JK184-induced growth suppression of BCa cells. Mechanically, autophagy initiation is induced by JK184-mediated inhibition of the Akt/mTOR pathway. Notably, JK184-induced autophagy plays a protective role, as blocking autophagy enhances the antitumor activity of JK184 in BCa cells. In summary, our findings suggest a novel mechanism of JK184-induced autophagy, which may provide a potential therapeutic strategy against BCa by targeting autophagy simultaneously.

## 2. Materials and Methods

### 2.1. Cell Culture

Human breast cancer cell lines (BT549 (ATCC), SKBR3 (ATCC), MCF-7 (ATCC), MDA-MB-231 (ATCC), and MDA-MB-468 (ATCC)) and normal epithelial breast cell line MCF-10A (ATCC) were cultured according to the ATCC guidelines and used within 6 months. Cells were maintained in RPMI1640 or DMEM with 10% fetal bovine serum (Thermo Fisher Scientific), 100 mg/mL streptomycin (Millipore Sigma), and 100 *μ*/mL penicillin (Millipore Sigma, Burlington, MA, USA), in a humidified 37°C incubator under 5% CO_2_ atmosphere.

### 2.2. Reagents

JK184 (HY-13307), 3-methyladenine (3-MA) (HY-19312), and ferrostatin-1 (HY-100579) were purchased from MedChem Express (USA). z-VAD-fmk (V116), DMSO (D2650), crystal violet (C0775), and chloroquine (CQ) diphosphate salt (C6628) were purchased from Millipore Sigma. 3-MA, MTT, Crystal Violet, and CQ diphosphate salt were dissolved in PBS.

Antibodies used in this study are listed as follows: cleaved caspase-3 (9664S; Cell Signaling Technology, Danvers, MA, USA), caspase 3 antibody (#9662; Cell Signaling Technology), Beclin1 (3738; Cell Signaling Technology), ATG5 (12994S; Cell Signaling Technology), Akt (4685; Cell Signaling Technology), phosphorylated (p-) Akt (Ser473) (4060; Cell Signaling Technology), mTOR (2972; Cell Signaling Technology), p-mTOR (Ser2448) (2971; Cell Signaling Technology), p70S6K (9202; Cell Signaling Technology), p-p70S6K (Ser371) (9208; Cell Signaling Technology), 4EBP1 (9452; Cell Signaling Technology), p-4EBP1 (Ser65) (9451; Cell Signaling Technology), PARP (ab74290; Abcam, Cambridge, MA, UK), p62 (sc-48402; Santa Cruz Biotechnology, Dallas, TX, USA), *β*-actin (sc-1616; Santa Cruz Biotechnology), LC3 (NB100-2220; Novus, Saint Charles, MO, USA), and horseradish peroxidase- (HRP-) conjugated anti-rabbit/mouse secondary antibody (Santa Cruz, sc-2004/sc-2005). Goat anti-mouse/rabbit Alexa Fluor 488 and goat anti-mouse/rabbit Alexa Fluor 594 (Thermo Fisher Scientific) were used for immunofluorescence.

### 2.3. MTT Assay

The growth of JK184-treated BCa cells was assessed using the 3-(4,5-dimethylthiazol-2-yl)-2,5-diphenylte-trazolium bromide (MTT; Sigma) assay. Cells were plated in 96-well cell culture plates (4 × 10^3^ cells/well) and treated with an indicated concentration of JK184 for 24 h. 20 *μ*l MTT (5 mg/ml) was added to each well. After 4 h incubation, the medium was removed and 150 *μ*l DMSO was added to each well to dissolve the crystal formazan dye. An enzyme-linked immunosorbent assay reader was used to measure the absorbance at 570 nm. The value of absorbance in each group was normalized to that in the control group. This experiment was repeated three times, with 5 samples per group.

### 2.4. EdU Labeling Assay

EdU incorporation was examined by using the EdU Cell Proliferation Assay Kit (Ribobio, C10310-3). Briefly, cells were plated in 96-well cell culture plates (4 × 10^3^ cells/well) and treated with the indicated treatments. Then, the cells were incubated with 10 *μ*M EdU for 2 hours at 37°C. After incubation, the cells were incubated with 100 *μ*L of the Triton X-100-based permeabilization regent for 20 minutes. After washes, 100 *μ*L dye reaction solution was added and incubated for 30 minutes at room temperature in the dark. Subsequently, DAPI was used for nuclear staining, followed by imaging with a DM2500 fluorescence microscope (Leica). This experiment was repeated three times, with 3 samples per group.

### 2.5. Colony Formation Assay

Cells were seeded in 24-well cell culture plates (200 cells/well) and continuously cultured for 14 days. Meanwhile, cells were subjected to the indicated treatments. Clones were stained with crystal violet for 15 minutes and washed three times. The Molecular Imager Gel Do XR + System (BIO-RAD) and Image J software (NIH) were used to photograph or count the visible colonies. This experiment was repeated three times.

### 2.6. Trypan Blue Staining

Cells were plated in 6-well cell culture plates (5 × 10^5^ cells/well) and treated with different concentrations of JK184 (0, 4, and 8 nM) for 24 h. Then, the cells were stained with trypan blue (0.4%), and the number of viable cells that have a clear cytoplasm (viable cells) was calculated. This experiment was repeated three times, with 3 samples per group.

### 2.7. Acridine Orange Staining

Cells were subjected to the corresponding treatments for 24 hours and then stained with 1 *μ*M acridine orange (Sigma-Aldrich) in 5% FBS-PBS solution at 37°C for 15 minutes. Cells were washed with PBS, and then confocal laser scanning microscopy was used to visualize images (Carl Zeiss Microimaging). This experiment was repeated three times.

### 2.8. Immunofluorescence

Cells were planted onto glass cover slips in 24-well culture plates for at least 12 hours. After being treated with corresponding treatments, cells were fixed with 4% paraformaldehyde at room temperature for more than 30 mins. After washing three times with PBS, cells were blocked and punched with 0.2% Triton X-100 (Sigma-Aldrich, 9002-93-1) and 5% goat serum (Sigma-Aldrich, G9023) for 1 hour. Then, the cells were incubated with primary antibody at 4°C overnight. Subsequently, the cells were washed with PBS and then incubated with secondary antibody (DyLight 488-conjugated goat anti-rabbit IgG and DyLight 594-conjugated goat anti-mouse IgG) at 37°C for 1 h. Nuclei were finally stained with DAPI for 10 min. Confocal laser scanning microscopy (Carl Zeiss Microimaging) was used to visualize images. The experiment was repeated three times.

### 2.9. Short Hairpin RNA Construction

ATG5, Beclin1, and negative control (NC) shRNA were synthesized by Genephama. 2 *μ*g shRNA was transfected into cells by using Lipofectamine 2000 (Invitrogen), following the manufacturer's instruction. The shRNA sequences are as follows: 5' GAC GUU GGU AAC UGA CAA ATT 3' (sh*Atg5*); 5' GGA GCC AUU UAU UGA AAC UTT 3' (sh*Beclin1*).

### 2.10. Immunoblotting

Cells were washed with normal saline and suspended in RIPA buffer (1.0 mM EDTA, 50 mM Tris, 0.1% SDS, 150 mM NaCl, 1% Triton X-100, 1 mM PMSF, and 1% sodium deoxycholate) for 30 min on ice to gain extract. Then, the proteins were separated in 12% SDS PAGE. After being transferred to the PVDF membrane, nonspecific binding was blocked with 5% nonfat dry milk followed by incubation with the appropriate primary antibodies overnight. Membranes were washed with 1 × TBS-T, and blots were incubated with secondary antibodies at 37°C for 2 h. Bound antibodies were detected by using the HRP immunoblotting detection reagent. The experiment was repeated three times.

### 2.11. Statistical Data Analysis

GraphPad Prism 6.0 software was used to analyze significant differences between the 2 groups. Comparisons between the two groups were performed by two-tailed Student's *t*-test or one-way ANOVA. Comparisons of repeated measurements over time were performed using two-way ANOVA. Data were displayed as means ± s. d., and significance is described as follows: ^*∗*^, *P* < 0.05; ^*∗∗*^, *P* < 0.01; ^*∗∗∗*^, *P* < 0.001.

## 3. Results

### 3.1. JK184 Inhibits Proliferation of BCa Cells

**Figure 1:**
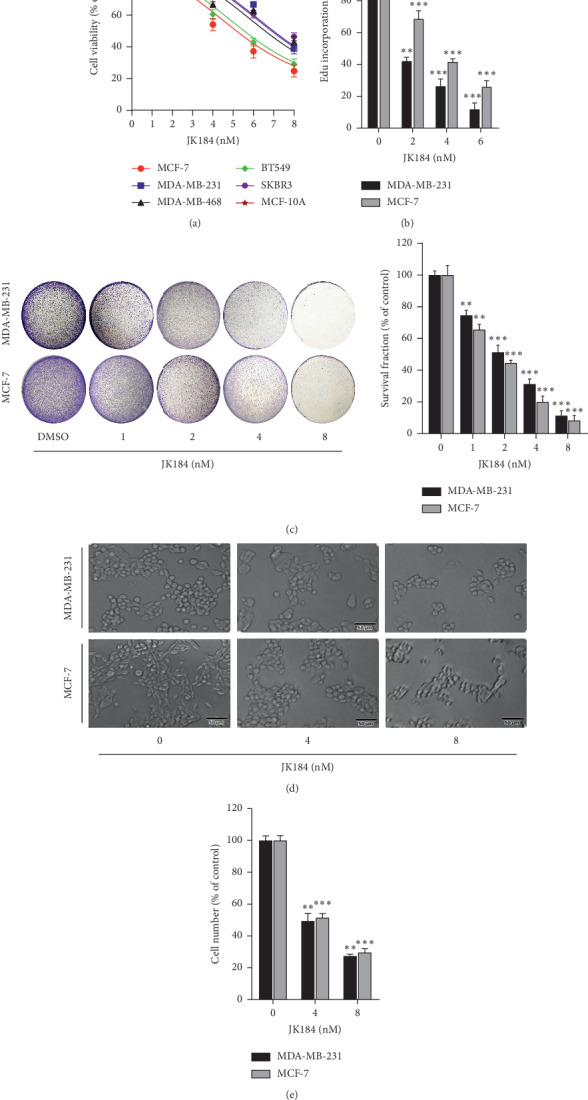
JK184 inhibits the growth of BCa cells. (a) JK184 inhibited BCa cell growth. Cell growth was measured in MCF-7, MDA-MB-231, MDA-MB-468, BT549, SKBR3, and MCF-10A cells treated with the indicated concentrations of JK184 for 24 hours. (b) JK184 inhibited BCa cell proliferation measured by EdU labeling. (c) JK184 suppressed colony formation in BCa cells. Cells (200 cells/well) were cultured in the indicated concentrations of JK184 for 2 days and continuously cultured in a fresh medium for another 12 days. Representative images (left) and survival fraction were shown (right). (d) The status of cells that were treated with JK184 in different concentrations. Scale bar, 50 *μ*m. (e) The number of cells that have a clear cytoplasm (viable cells) was counted after being treated with indicated concentrations of JK184 for 24 hours. ^*∗*^, *P* < 0.05; ^*∗∗*^, *P* < 0.01; ^*∗∗∗*^, *P* < 0.001.

To verify the antitumor effect of JK184 on BCa, various of BCa cell lines, including MCF-7, MDA-MB-231, MDA-MB-468, BT549, and SKBR3, and a normal breast epithelial cell line MCF-10A were treated with JK184. As shown in Figures 1(a) and S1A, B, the cell growth of all examined breast cancer cells was inhibited by JK184 in 24 hours, while MCF-10A cells showed higher tolerance to JK184. Additionally, EdU labeling (Figure 1(b)) and colony formation assay (Figure 1(c)) revealed that JK184 treatment resulted in marked proliferation inhibition in BCa cells. Consistently, decreased living cell numbers were observed by using a microscope (ZEISS) (Figure 1(d), Figure S1C, D) and Trypan blue staining (Figure S1E) in JK184-treated BCa cells. Taken together, these data reveal that JK184 shows a significant antitumor effect in BCa cells.

### 3.2. JK184 Induces Autophagy in BCa Cells

**Figure 2:**
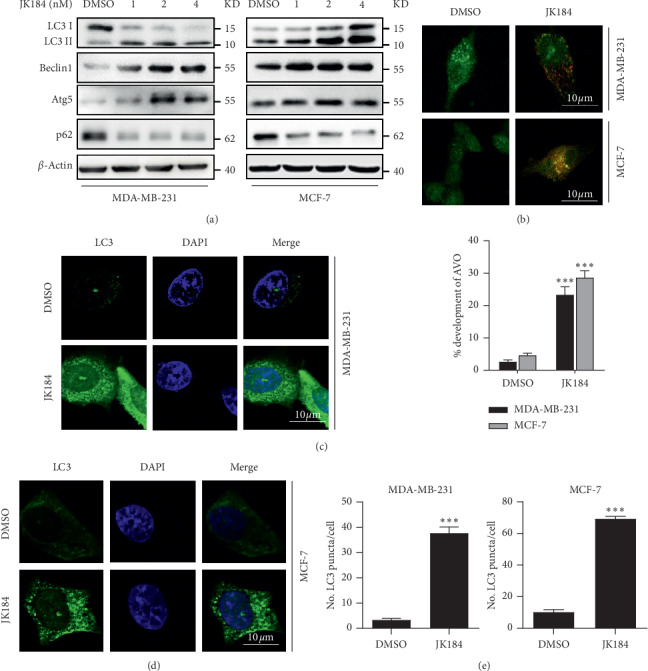
JK184 induces autophagy in BCa cells. (a) Western blot analysis of LC3, Beclin 1, Atg5, and p62/SQSTM1 in cells treated with the indicated concentrations of JK184 for 24 hours. (b) (Above) autophagy measured by acridine orange staining of cells treated with or without 4 nM JK184 for 24 hours. (Below) the total number of acidic vesicular organelles (AVO) per cell. Scale bar, 10 *μ*m. (c-d) Endogenous LC3 puncta formed in BCa cells treated with DMSO or 4 nM JK184 for 24 hours. Scale bar, 10 *μ*m. (e) Total number of endogenous LC3 puncta per cell.

Growing evidence indicated that autophagy was promoted in response to anticancer therapies, especially drug treatment [12]. To clarify whether JK184 induced autophagy in BCa cells, we explored the expression of the autophagy classical marker LC3B (the conversion of LC3B-I to lipidated LC3B-II indicated autophagy occurrence), autophagy-related proteins (Atg5, Beclin 1), and autophagy substrate p62/SQSTM1. As shown in Figures 2(a) and S2, JK184 induced marked autophagy induction, as evidenced by increased LC3B-II conversion, protein expression of Atg5 and Beclin 1, and decreased protein expression of p62/SQSTM1, in a dose-dependent manner. BCa cells were stained by acridine orange to evaluate the induction of autophagy. Bountiful cytoplasmic acidic vesicular organelles (AVO) were promptly examined in JK184-treated BCa cells (Figure 2(b)). Furthermore, the endogenous LC3B puncta arose following JK184 treatment in BCa cells (Figures 2(c)–2(e)). Conversely, 3-MA and CQ, the autophagy inhibitors, prominently decreased LC3B-II in JK184-treated BCa cells (Figure S3). Together, our results demonstrate that JK184 promotes the autophagosome formation in BCa cells.

### 3.3. JK184 Promotes the Autophagy Flux in BCa Cells

**Figure 3:**
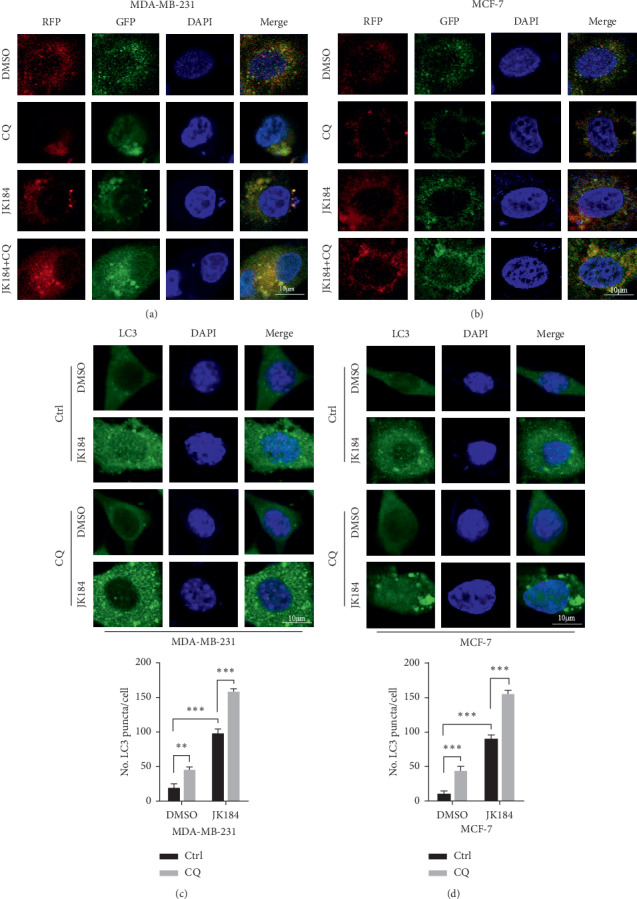
JK184 promotes autophagy flux in BCa cells. (a-b) Cells were transiently transfected with an RFP-GFP tandem fluorescent-tagged LC3 (RFP-GFP-LC3). Furthermore, cells were treated with 4 nm JK184 or in combination with 10 *μ*M chloroquine (CQ) for 24 hours. Scale bar, 10 *μ*m. (c-d) (Above) endogenous LC3 puncta analyzed by immunofluorescence in cells treated as in (a). (Below) total number of endogenous LC3 puncta per cell. ^*∗∗*^, *P* < 0.01; ^*∗∗∗*^, *P* < 0.001. Scale bar, 10 *μ*m.

We next detected whether JK184 promoted the autophagy flux in BCa cells. A tandem mRFP-GFP tagged LC3 plasmid was used in JK184-treated BCa cells. We found that more autolysosomes than autophagosomes were formed under JK184 treatment (GFP^−^RFP^+^ and GFP^+^RFP^+^ signal represented autolysosomes and autophagosomes, respectively) (Figures 3(a)–3(b)). LC3B lipidation was further increased by combinatorial treatment with both JK184 and autolysosome inhibitor (CQ) (Figures 3(c)-3(d) and S3). Moreover, the immunofluorescence analysis results of colocalized LC3B and LAMP2 in BCa cells demonstrated that the colocalization of autophagosome and lysosome was more obvious after being treated with JK184 (Figure S4). Thus, these findings reveal that JK184 triggers complete autophagy flux in BCa cells.

### 3.4. Blocking Autophagy Enhances JK184-Induced Growth Inhibition in BCa Cells

**Figure 4:**
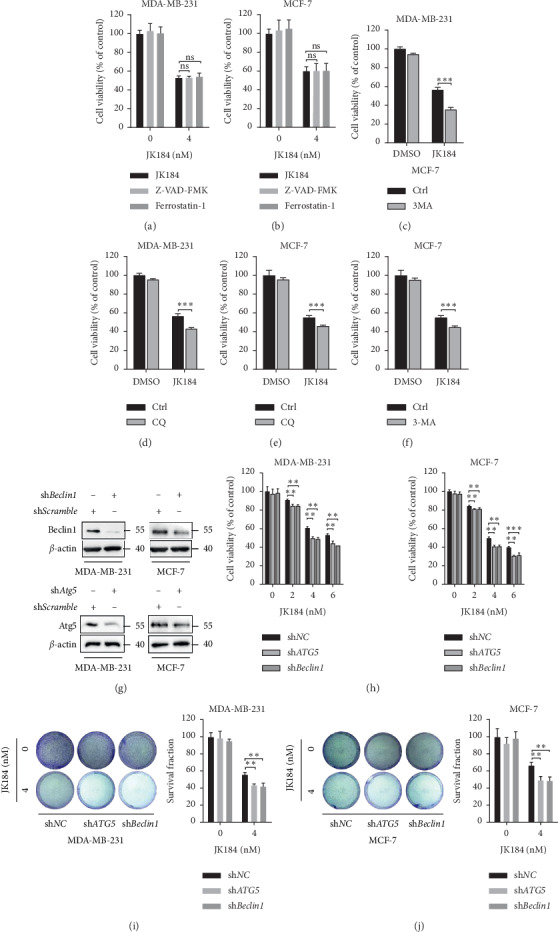
Inhibition of autophagy promotes the antiproliferative effect of JK184 in BCa cells. (a-b) JK184 inhibited the growth of BCa cells independent of apoptosis and ferroptosis. Cells were treated with DMSO, Z-VAD-FMK (20 *μ*M), or ferrostatin-1 (1 *μ*M) in the presence or absence of JK184 (4 nM) for 24 hours. (c–f) Cells were treated with DMSO, chloroquine (CQ), or 3-methyladenine (3-MA) in the presence or absence of JK184 (4 nM) for 24 hours. (g) Cells were transfected with shRNA against Atg5 or Beclin 1 or control (50 nM) for 48 h and then treated with JK184 at 4 nm for another 24 hours. (h) Cell viability of BCa cells transferred with shATG5 or shBeclin1 followed by treatment with an indicated concentration of JK184. (i-j) Colony formation assay of BCa cells transferred with shATG5 or shBeclin1 followed by treatment with 4 nM JK184. Representative images (left) and survival fraction were shown (right). ns, *P* > 0.05; ^*∗∗*^, *P* < 0.01.

To demonstrate whether apoptosis or ferroptosis was involved in JK184-mediated growth inhibition, JK184 treatment combined with Z-VAD-FMK (a pan-caspase inhibitor) or ferrostatin-1 (a ferroptosis inhibitor) was conducted. As shown in Figures 4(a)-4(b), neither the apoptosis inhibitor nor the ferroptosis inhibitor had an obvious effect on JK184-induced growth inhibition in BCa cells. Also, the levels of the markers of apoptosis, such as poly (ADP-ribose) polymerase (PARP) and cleaved-caspase 3 [13], and the key molecule of ferroptosis cystine-glutamate antiporter (xCT) [14] did not change obviously after being treated with JK184 (Figure S5). To further confirming what role autophagy played in JK184-induced cell toxicity in BCa cells, we used various autophagy inhibitors in JK184-treated BCa cells. As shown in Figures 4(c)–4(f), CQ and 3-MA led to remissive cell viability. Consistently, knockdown of *ATG5* and *BECN1* further enhanced JK184-induced growth inhibition (Figures 4(g)–4(j)). Thus, these data suggest that autophagy exerts cytoprotective effects during JK184 treatment and blocking autophagy enhances the antitumor effect of JK184 in BCa cells.

### 3.5. JK184 Induces Autophagy via Inhibiting the Akt/mTOR Pathway in BCa Cells

**Figure 5:**
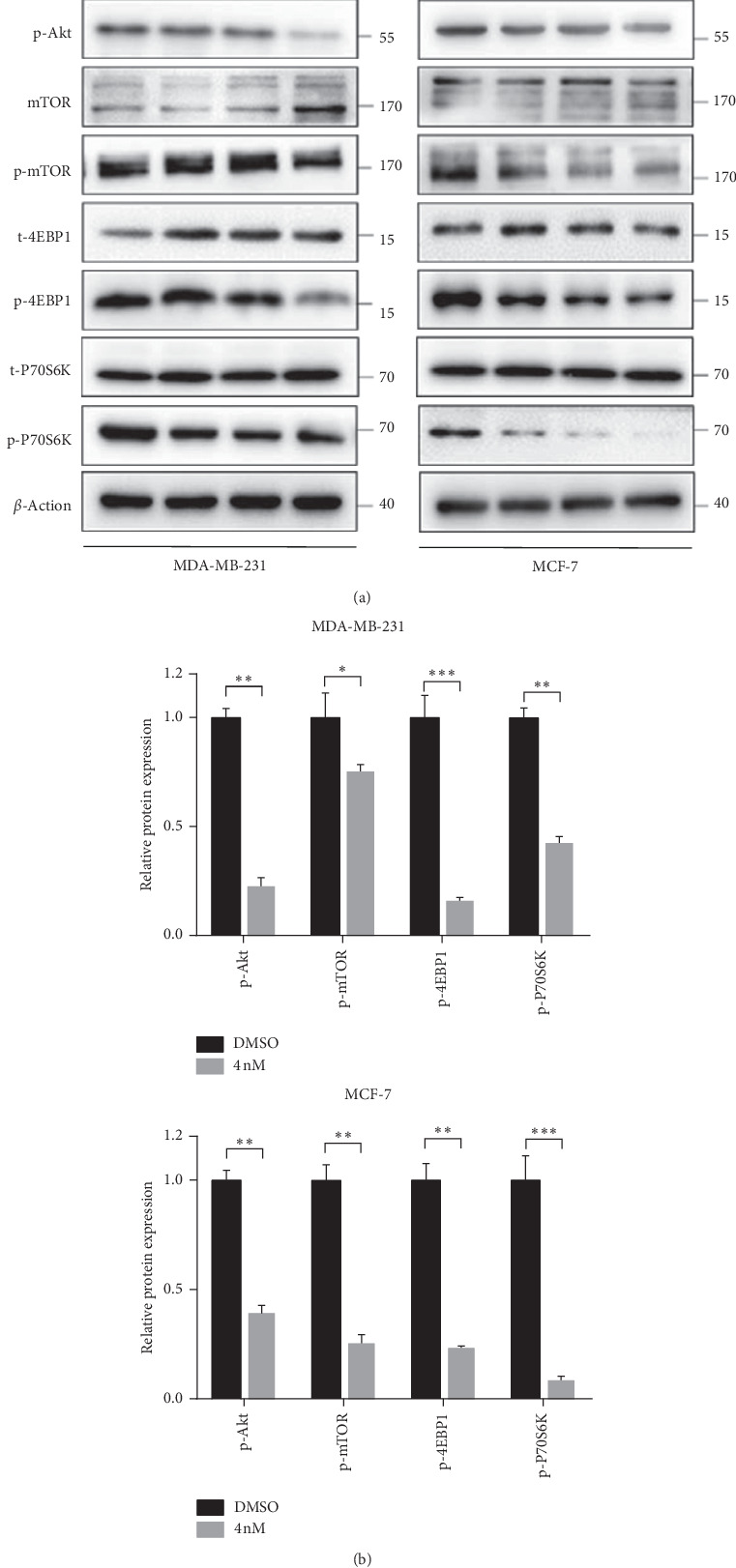
JK184 induces autophagy by inhibiting the Akt/mTOR pathway. (a) Immunoblot analysis of phosphorylation of Akt, mTOR, p70S6K, and 4EBP1 in cells treated with the indicated concentrations of JK184 for 24 hours. The expression levels of total Akt, mTOR, p70s6k, and 4EBP1 were used as internal controls. (b) Densitometry quantification of the band intensities in A (DMSO- and 4 nM JK184-treated groups) was carried out using ImageJ software and is presented by normalizing the values of the DMSO group to 1, relative to the corresponding *β*-actin.

There are many evidences indicating that the PI3K/Akt signaling pathway is involved in the occurrence of BCa, and Akt/mTOR is a classic signaling for inhibition of autophagy [15]. To further explore whether the Akt/mTOR pathway was involved in JK184-induced autophagy, we examined the levels of Akt/mTOR pathway-related proteins, including total or phosphorylated Akt, mTOR, p70S6K, and 4EBP1. We found decreased protein phosphorylation of Akt, mTOR, p70S6K, and 4EBP1 in JK184-treated cells (Figure 5). The results demonstrate that the inhibition of the Akt/mTOR pathway is responsible for JK184-induced autophagy in BCa cells.

## 4. Discussion

JK184 is reported to antagonize the Hedgehog pathway and inhibit glioma-dependent transcriptional activity [16]. It functions by efficiently, rapidly, and reversibly interfering microtubules depolymerization and microtubule-dependent signalling events [17]. In this study, our data showed that JK184 exhibited potent antitumor effects on BCa cells. In addition, we revealed a previously unreported mechanism by which JK184 markedly induced autophagy via repressing the Akt/mTOR pathway. Subsequently, we identified JK184-mediated autophagy as a protective role, evidenced by the promotion of the antiproliferative effect of JK184 following inhibition of autophagy in BCa cells.

Same as pancreatic cancer, in this study, our results indicated that the growth of BCa cells can also be inhibited by JK184. The data showed that JK184 can reduce the cell viability of multiple BCa cells, including the high metastatic potential BCa cell line (MDA-MB-231). A more in-depth study of the mechanism about JK184 inhibiting the growth of BCa cells may provide the possibility of JK184 in the BCa clinical treatment.

Generally, autophagy is proposed as an evolutionarily conserved mechanism that promotes cancer cell survival by recycling nutrients to furnish their proliferation. In addition, autophagy has been found to prevent aging, metabolic disorders, infections, and neurodegenerative diseases. Growing evidence indicates the key role of autophagy in cancer therapy. It has been reported that autophagic cell death induced by certain drugs significantly suppresses tumor growth, especially in the apoptosis-deficient cancer types. For example, a previous study suggested that ivermectin blocked the PAK1/Akt axis, therefore inducing cytostatic autophagy in BCa [18]. However, the prosurvival property of autophagy induced by cancer therapy is also reported to lead to chemoresistance in part. Accordingly, it is of importance to figure out the role of autophagy in a particular context to manipulate autophagy for chemotherapy benefit.

Here, we reported that JK184 induced autophagic flux, which impaired the growth suppression effect of JK184 in BCa cells. Also, inhibition of the autophagic flux induced by JK184 can significantly enhance the growth suppression effect. This observation indicates that autophagy functions as a prosurvival mechanism to reduce the cytotoxicity of JK184, suggesting combinational use of autophagy inhibitors might be a potential strategy to enhance the antitumor activity of JK184. In agreement with our results, a plenty of small molecule inhibitors targeting cancer growth inhibition unexpectedly induced protective autophagy against the anticancer effect of these drugs. For instance, osimertinib, an EGFR inhibitor, was reported to work modestly in colorectal cancer due to induction of protective autophagy mediated by the MCT1/LKB1/AMPK axis [19]. A recent report also showed that the blockage of ER-phagy significantly promoted the anticancer effect of the ALK inhibitor brigatinib in ALK-negative cancer cells, revealing that combinational treatment with an autophagy inhibitor might be an actionable strategy for improving cancer therapy efficacy [20]. These previous reports support our research that JK184-induced autophagy may be implicated as a cause of resistance to therapies in BCa. Accordingly, the role of autophagy could have opposite impacts on cancer cells: it suppresses tumor growth in a condition but relieves therapeutic effect of cancer cells in another, indicating that the underlying role of autophagy can guide the treatment direction to some extent.

There are several signal pathways involved in the activation of autophagy. As the most classic autophagy-related signal pathway, the AKT/mTOR pathway plays an important role in drug-induced protective autophagy. Apatinib, an inhibitor of vascular endothelial growth factor receptor-2, has been reported to induce both protective autophagy and apoptosis in human ATC cells via the AKT/mTOR pathway [21]. 3'-epi-12*β*-Hydroxyfroside (HyFS) is a new cardenolide that can inhibit cell proliferation of lung cancer cells. Study has demonstrated that HyFS can block the AKT/mTOR pathway through ubiquitin-mediated degradation of Hsp90, leading to a cytoprotective autophagy [22]. As shown in our data, JK184 is similar to these anticancer drugs, and it can suppress the activation of the AKT/mTOR pathway, which may promote the protective autophagy.

In summary, our finding suggests that JK184 has a significant antitumor effect in BCa cells. Meanwhile, JK184 restrains the phosphorylation of AKT/mTOR, which, in turn, results in the initiation of autophagy. We also show that autophagy contributes to the resistance of BCa to JK184 treatment, and the combinatorial use of JK184 with autophagy inhibitors might be a potent anticancer therapeutic strategy.

## Data Availability

All data and materials are available upon request from the corresponding author.

## Supplementary Materials

Supplementary Materials Supplementary figure 1 (figure S1): the cell viability of BCa cells after being treated with JK184. Supplementary figure 2 (figure S2): the alteration of autophagy-related protein induced by JK184 in BCa cells. Supplementary figure 3 (figure S3): CQ and 3-MA inhibit JK184-induced autophagy. Supplementary figure 4 (figure S4): autophagosome and lysosome colocalization in BCa cells. Supplementary figure 5 (figure S5): the levels of apoptosis- and ferroptosis-related protein markers in BCa cells after being treated with JK184.

## References

[1] Chen W., Zheng R., Baade P. D. (2016). Cancer statistics in China, 2015. *CA: A Cancer Journal for Clinicians*.

[2] Jemal M. L., Sprunck-Harrild K., Ruddy K. J. (2015). Study protocol for young & strong: a cluster randomized design to increase attention to unique issues faced by young women with newly diagnosed breast cancer. *BMC Public Health*.

[3] Meyer N., Gnant M. (2017). Breast cancer. *The Lancet*.

[4] Galluzzi L., Pietrocola F., Levine B., Kroemer G. (2014). Metabolic control of autophagy. *Cell*.

[5] Sui X., Chen R., Wang Z. (2013). Autophagy and chemotherapy resistance: a promising therapeutic target for cancer treatment. *Cell Death & Disease*.

[6] Han B., Klionsky D. J. (2017). Autophagy wins the 2016 nobel prize in physiology or medicine: breakthroughs in baker’s yeast fuel advances in biomedical research. *Proceedings of the National Academy of Sciences*.

[7] Mizushima N. (2007). Autophagy: process and function. *Genes & Development*.

[8] Mizushima N., Yoshimori T., Ohsumi Y. (2011). The role of Atg proteins in autophagosome formation. *Annual Review of Cell and Developmental Biology*.

[9] Zhou J., Li G., Zheng Y. (2015). A novel autophagy/mitophagy inhibitor liensinine sensitizes breast cancer cells to chemotherapy through DNM1L-mediated mitochondrial fission. *Autophagy*.

[10] Huang X., Wei S., Zhao Y. (2017). Anti-proliferation of breast cancer cells with itraconazole: hedgehog pathway inhibition induces apoptosis and autophagic cell death. *Cancer Letters*.

[11] Lei N., Liu S., Wang N. (2015). Biodegradable polymeric micelles encapsulated JK184 suppress tumor growth through inhibiting Hedgehog signaling pathway. *Nanoscale*.

[12] Liu R. K., Yu D., Lum J. J. (2007). Autophagy inhibition enhances therapy-induced apoptosis in a Myc-induced model of lymphoma. *Journal of Clinical Investigation*.

[13] Thomas-Tikhonenko S., Takimoto T., Hemler M. E. (2019). Integrin-independent support of cancer drug resistance by tetraspanin CD151. *Cellular and Molecular Life Sciences*.

[14] Liu T., Jiang L., Tavana O., Gu W. (2019). The deubiquitylase OTUB1 mediates ferroptosis via stabilization of SLC7A11. *Cancer Research*.

[15] Kondo Y., Kanzawa T., Sawaya R., Kondo S. (2005). The role of autophagy in cancer development and response to therapy. *Nature Reviews Cancer*.

[16] Lee J., Wu X., Pasca di Magliano M. (2007). A small-molecule antagonist of the hedgehog signaling pathway. *ChemBioChem*.

[17] Hebrok T., Rack P. G., Firestone A. J. (2009). The imidazopyridine derivative JK184 reveals dual roles for microtubules in Hedgehog signaling. *Angewandte Chemie International Edition*.

[18] Dou Q., Chen H.-N., Wang K. (2016). Ivermectin induces cytostatic autophagy by blocking the PAK1/akt axis in breast cancer. *Cancer Research*.

[19] Lan P., Jiang J., Xie N. (2019). MCT1 relieves osimertinib-induced CRC suppression by promoting autophagy through the LKB1/AMPK signaling. *Cell Death & Disease*.

[20] Qin Z., Gao W., Zhou L. (2019). Repurposing brigatinib for the treatment of colorectal cancer based on inhibition of ER-phagy. *Theranostics*.

[21] Feng H., Cheng X., Kuang J. (2018). Apatinib-induced protective autophagy and apoptosis through the AKT-mTOR pathway in anaplastic thyroid cancer. *Cell Death & Disease*.

[22] Sun Y., Huang Y.-H., Huang F.-H. (2018). 3′-epi-12*β*-hydroxyfroside, a new cardenolide, induces cytoprotective autophagy via blocking the Hsp90/Akt/mTOR axis in lung cancer cells. *Theranostics*.

