# Supplementary material for: Inhibition of JK184-Induced Cytoprotective Autophagy Potentiates JK184 Antitumor Effects in Breast Cancer
Source: J Oncol. 2020 Jun 2;2020:1657896. doi: 10.1155/2020/1657896 (PMC7288248; doi:10.1155/2020/1657896)
Supplement: Supplementary Materials — Supplementary figure 1 (figure S1): the cell viability of BCa cells after being treated with JK184. Supplementary figure 2 (figure S2): the alteration of autophagy-related protein induced by JK184 in BCa cells. Supplementary figure 3 (figure S3): CQ and 3-MA inhibit JK184-induced autophagy. Supplementary figure 4 (figure S4): autophagosome and lysosome colocalization in BCa cells. Supplementary figure 5 (figure S5): the levels of apoptosis- and ferroptosis-related protein markers in BCa cells after being treated with JK184. [file 1657896.f1.docx]

**Supplementary Figure 1.**


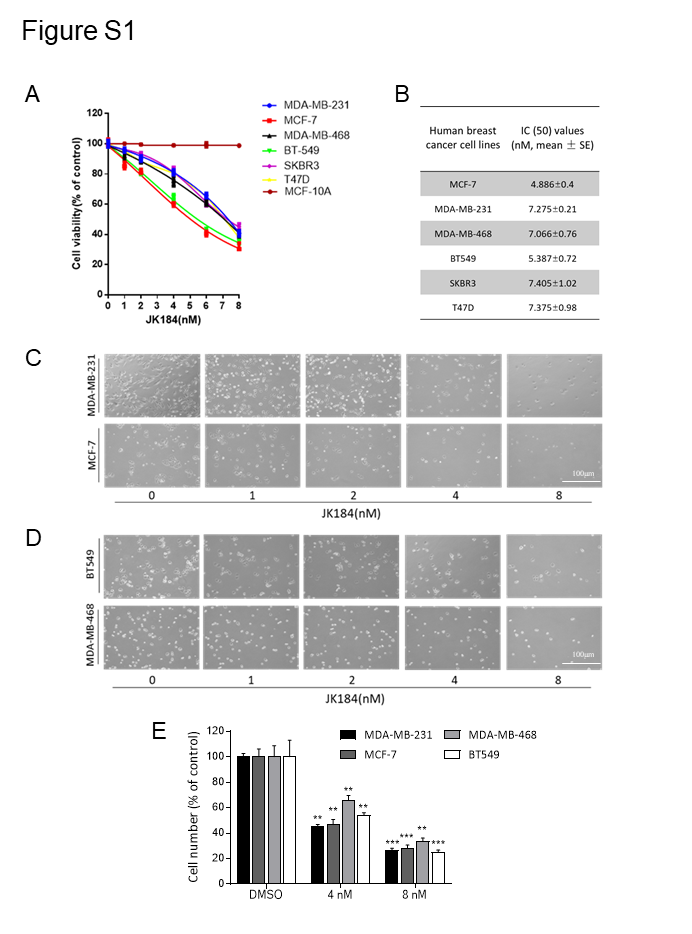


**Figure. S1. JK184 inhibit the cell viability of BCa cells.** A, MTT assay of various BCa cell lines treated with the indicated concentrations of JK184 for 24 hours. B, the precise IC50 of JK184 in different BCa cells. Calculated from MTT experiment results. C-D, the status of cells that treated with different concentration JK184. Scale bar, 100 μm. E, Trypan blue staining to calculate the number of cells that have clear cytoplasm (viable cells), after being treated with the indicated concentrations of JK184 for 24 hours.

**Supplementary Figure 2.**


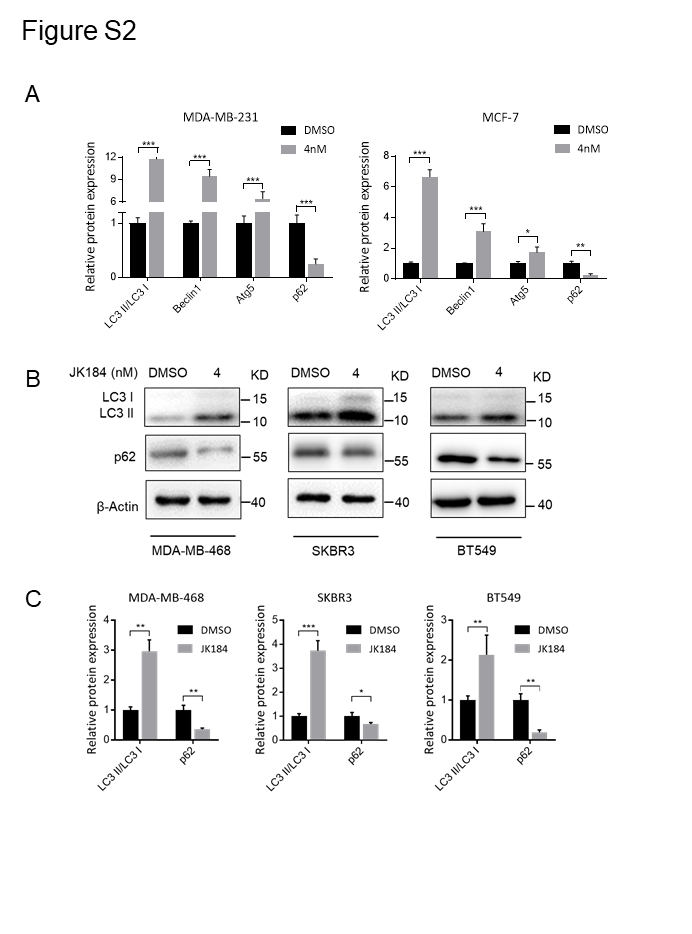


**Figure S2. JK184 leads to the alterations of autophagy related protein.** A, densitometry quantification of the band intensities in **Fig. 2A** (DMSO and 4 nM JK184 treated groups) was carried out using ImageJ software and is presented as a percentage of relative densitometry normalized to β-actin. B, immunoblot analysis of LC3 and p62 in MDA-MB-468, SKBR3 and BT549 cells treated with the indicated concentrations of JK184 for 24 hours. C, densitometry quantification of the band intensities in **B** (DMSO and 4 nM JK184 treated groups) was carried out using ImageJ software and is presented by normalizing the values of DMSO group to 1, relative to the corresponding β-actin.

**Supplementary Figure 3.**

**
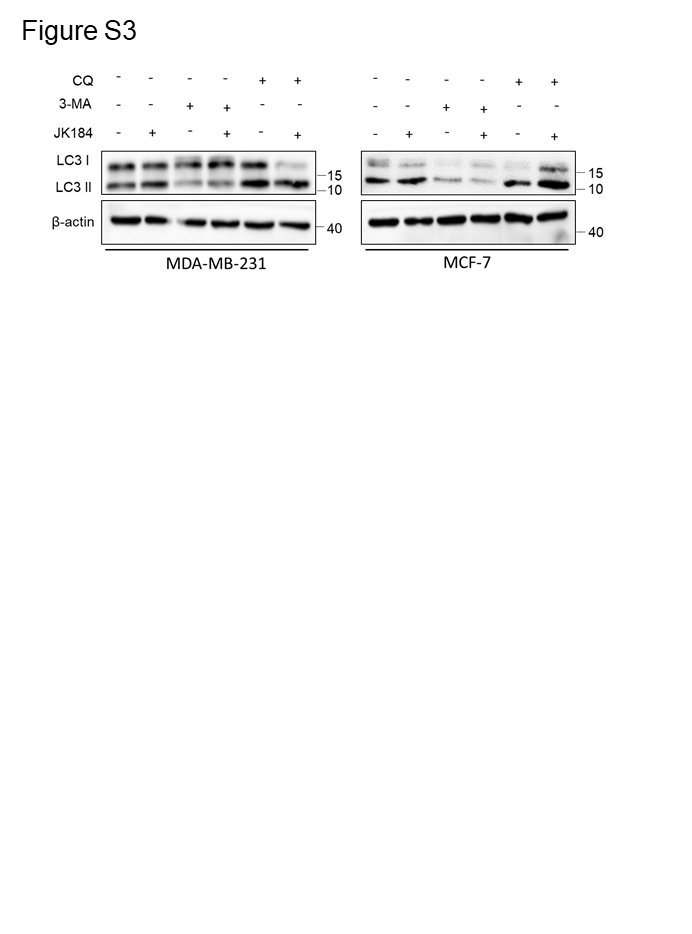
**

**Figure S3. CQ and 3-MA inhibit JK184 induced autophagy.** Immunoblot analysis of LC3 in cells treated with JK184 and autophagy inhibitors (CQ and 3-MA) for 24 hours.

**Supplementary Figure 4.**

**
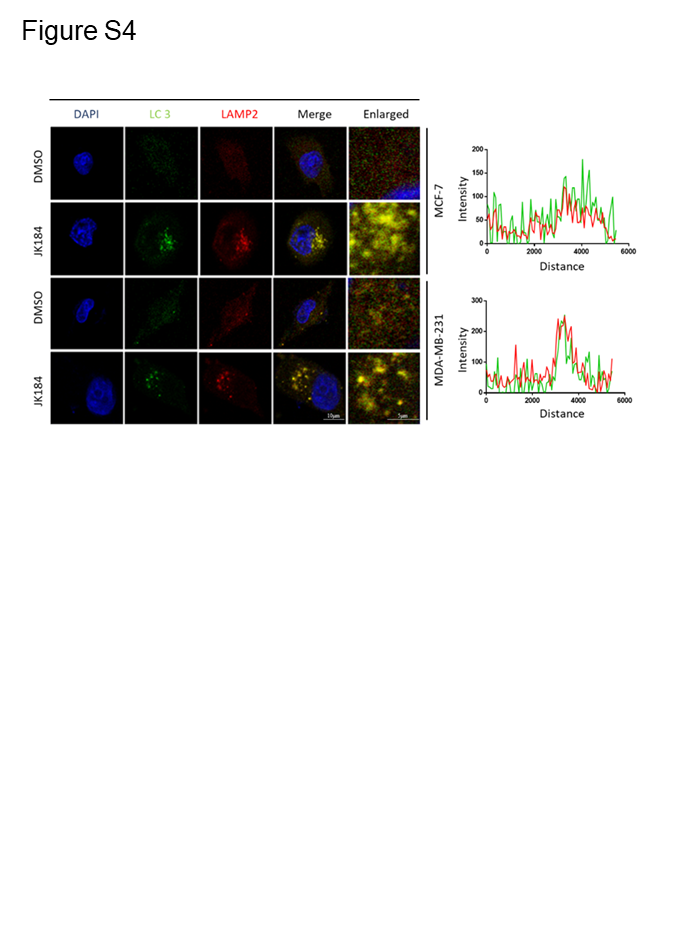
**

**Figure S4. JK184 induces the fusion of autophagosome and lysosome in BCa cells.** Immunofluorescence analysis of colocalized LC3 and LAMP2 in breast cancer cells treated with or without 4 nM JK184 for 24 hours. Scare bar, 10μm.

**Supplementary Figure 5.**


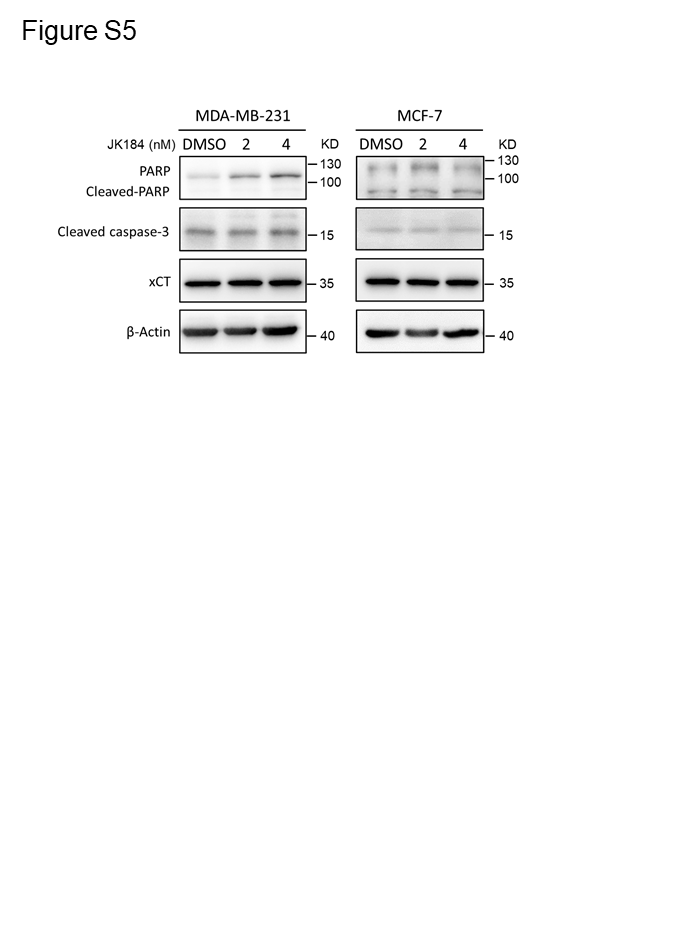


**Figure S5.** **JK184 treatment does not significantly induce apoptosis and ferroptosis in BCa cells.** Immunoblot analysis of total and cleaved PARP, cleaved caspase-3 and xCT in cells treated with the indicated concentrations of JK184 for 24 hours.
